# Genome assembly using Nanopore-guided long and error-free DNA reads

**DOI:** 10.1186/s12864-015-1519-z

**Published:** 2015-04-20

**Authors:** Mohammed-Amin Madoui, Stefan Engelen, Corinne Cruaud, Caroline Belser, Laurie Bertrand, Adriana Alberti, Arnaud Lemainque, Patrick Wincker, Jean-Marc Aury

**Affiliations:** Commissariat à l’Energie Atomique (CEA), Institut de Génomique (IG), Genoscope, BP5706, 91057 Evry, France; Université d’Evry Val d’Essonne, UMR 8030, CP5706, 91057 Evry, France; Centre National de Recherche Scientifique (CNRS), UMR 8030, CP5706, 91057 Evry, France

**Keywords:** Nanopore sequencing, Oxford nanopore, MinION**®** device, *de novo* genome assembly, Genome finishing

## Abstract

**Background:**

Long-read sequencing technologies were launched a few years ago, and in contrast with short-read sequencing technologies, they offered a promise of solving assembly problems for large and complex genomes. Moreover by providing long-range information, it could also solve haplotype phasing. However, existing long-read technologies still have several limitations that complicate their use for most research laboratories, as well as in large and/or complex genome projects. In 2014, Oxford Nanopore released the MinION® device, a small and low-cost single-molecule nanopore sequencer, which offers the possibility of sequencing long DNA fragments.

**Results:**

The assembly of long reads generated using the Oxford Nanopore MinION® instrument is challenging as existing assemblers were not implemented to deal with long reads exhibiting close to 30% of errors. Here, we presented a hybrid approach developed to take advantage of data generated using MinION® device. We sequenced a well-known bacterium, *Acinetobacter baylyi ADP1* and applied our method to obtain a highly contiguous (one single contig) and accurate genome assembly even in repetitive regions, in contrast to an Illumina-only assembly. Our hybrid strategy was able to generate NaS (Nanopore Synthetic-long) reads up to 60 kb that aligned entirely and with no error to the reference genome and that spanned highly conserved repetitive regions. The average accuracy of NaS reads reached 99.99% without losing the initial size of the input MinION® reads.

**Conclusions:**

We described NaS tool, a hybrid approach allowing the sequencing of microbial genomes using the MinION® device. Our method, based ideally on 20x and 50x of NaS and Illumina reads respectively, provides an efficient and cost-effective way of sequencing microbial or small eukaryotic genomes in a very short time even in small facilities. Moreover, we demonstrated that although the Oxford Nanopore technology is a relatively new sequencing technology, currently with a high error rate, it is already useful in the generation of high-quality genome assemblies.

**Electronic supplementary material:**

The online version of this article (doi:10.1186/s12864-015-1519-z) contains supplementary material, which is available to authorized users.

## Background

The technology of long-read sequencing now offers different alternatives to solve genome assembly problems (for example, in complex regions involving repeated elements or segmental duplications) and haplotype phasing, which cannot be resolved adequately by short-read sequencing. Application of the single-molecule real-time sequencing (SMRT) platform produced by Pacific Biosciences to small microbial as well as large complex eukaryotic genomes demonstrated the possibility of considerably improving genome assembly quality [1-4]. Microbial genome could now be fully assembled (at least in some cases) using Pacific Biosciences’s SMRT reads alone [2] or in combination with short but high quality reads [1]. The high error rate of SMRT reads renders the necessity for either deep coverage or a strategy of error correction using Illumina reads. It’s clear that the current yield and high cost per base of this technology remain a barrier for most genomic projects targeting large genomes. Moreover, the price of the commercially available Pacific Biosystems PacBio RS II instrument is high and the needs in terms of infrastructure and implementation does not make it accessible to the whole research community. Similar improvements in read length were also accomplished by the Illumina Truseq synthetic long-read sequencing strategy; its application to the human genome and the resolution of highly repetitive elements in the fly genome provided encouraging results [5,6] and showed the importance of long and high-quality reads. Nonetheless, the long range polymerase chain reaction step included in the library preparation may introduce important genome coverage biases. Moreover the time needed for library construction may be a limitation in a time-constrained project, and again does not make it accessible to the whole research community.

This year, Oxford Nanopore Technologies Ltd released the MinION® device, a single-molecule nanopore sequencer connected to a laptop through a USB 3.0 interface, to hundreds of members of the MinION® Access Programme (MAP) who are testing the new device. The technology is based on an array of nanopores embedded on a chip that detects consecutive 5-mers of a single-strand DNA molecule by electrical sensing [7]. This new technology provides several advantages: the MinION® device is small and low cost, the library construction involves a simplified method, no amplification step is needed, and data acquisition and analyses occur in real time. In the Oxford Nanopore technology, the two strands of a DNA molecule are linked by a hairpin and sequenced consecutively. When the two strands of the molecule are read successfully, a consensus is built to obtain a more accurate read (called 2D read). Otherwise only the forward strand sequence is provided (called 1D read).

MinION® tests were performed by all early access members, first on the phage lambda genome. Three recent publications of these studies [8-10] showed the production of long reads with an average size of 5,000 and 5,500 bp, respectively. These primary studies point to a high error rate in reads from the current version of MinION®. However, despite the high error rate, Ashton et al. [10] demonstrate the potential of the MinION® device for microbial sequencing. This motivated the need to develop new tools, either for MinION® read correction or for new alignment algorithms. Methods for correction of long reads produced for the Pacific Biosciences sequencer have already been proposed [1,11-13]. However, these methods are based on read alignment, thus the ability to correct input reads is linked to the local error rate. As a consequence, the size of the corrected read is closely correlated to the sequencing errors of the input long read. Long and relatively inaccurate reads that harbor hotspots of sequencing errors will lead to mosaic reads, with alternating regions of high and low fidelity. As existing assembly softwares were not implemented to deal with long reads with a high error rate, we developed a method based on a combination of two sequencing technologies: Oxford Nanopore and Illumina, to produce long and accurate synthetic reads before assembly.

## Results and discussion

### Overview of MinION® reads

**Figure 1:**
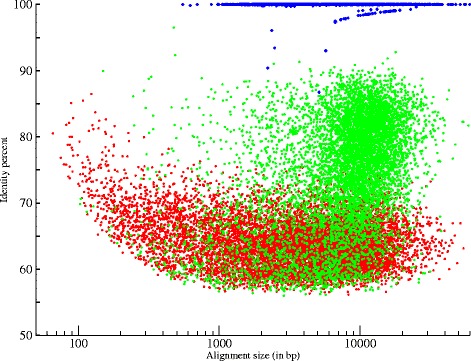
Comparison of MinION® and NaS reads quality. This plot shows the alignment identity and the alignment size of the MinION® 1D (red circles) and 2D (green circles) reads as well as NaS reads (blue circles). MinION® and NaS reads were aligned respectively using LAST [20] and BWA mem [25] softwares.

We performed five runs of MinION® sequencing with four different *A. baylyi* genomic DNA libraries (targeting two different mean fragment sizes: 8 kb and 20 kb), and two different flowcell chemistries, R7 and R7.3 (methods and Table 1). We produced a total of 66,492 reads, representing a genome coverage of approximately 57 ×. About 13% of these 66,492 reads were 2D reads, which represent 42% of the cumulative size, indicating a significant difference of length between 1D and 2D reads. The 1D reads had an average size of 2,052 bp, in contrast the average size of 2D reads reached 10,033 bp (Table 2). The N50 size is two times higher when using the 20 kb library, suggesting that we obtained longer MinION® reads when sheared size is increased. The lower average read size of run4 and run5 was due to a high proportion of very short 1D reads (< 500 bp). These two runs were achieved using the same library preparation (library4, Table 1). As previously reported [8-10], we observed a low mappability on the reference genome [14]; 83.2% of 2D reads and 16.6% of 1D reads were aligned (Figure 1 and Table 2). Thus, the real genome coverage, when only taking into account aligned nucleotides, is about 34 ×. The mean identity to the reference of 1D reads was 56.5% while 2D reads revealed a mean identity of 74.5%. The R7.3 chemistry showed several improvements in terms of throughput, proportion of 2D bases (greater than 42% with R7.3 and less than 27% with R7) and in quality of 2D reads (Additional file 1: Table S1). Even, if more recent chemistry and flowcells exhibited a significant progress, these first results still showed a heterogeneity in throughput and in proportion of 2D reads.

**Table 1:** **Overview of the five MinION® runs**

	**Run1**	**Run2**	**Run3**	**Run4**	**Run5**
DNA library	1	2	3	4	4
DNA fragment size	8 kb	20 kb	20 kb	20 kb	20 kb
Flowcell chemistry	R7	R7	R7.3	R7.3	R7.3
Number of reads	9,241	3,990	6,052	11,957	35,252
Cumulative size (Mb)	21.4	19.3	40.8	34.5	88.9
N50 size (bp)	5,388	11,288	10,217	12,729	13,967
Average size (bp)	2,314	4,830	6,746	2,886	2,523
% of 2D reads	6.5%	13.6%	43.3%	11.6%	9.7%
% of 2D bases	14.6%	27.1%	57.1%	42.7%	44.6%

**Table 2:** **Comparative summary statistics of the MinION® and corresponding NaS reads**

		**1D reads**	**2D reads**
MinION® reads			
aligned using LAST	# reads	57,911	8,581
	# reads (>10Kb)	3,609	3,866
	Cumulative size (Mbp)	118.9	86.1
	Average size (bp)	2,052	10,033
	N50 size (bp)	11,058	12,141
	Max size (bp)	123,135	58,704
	Aligned reads	9,623 (16.6%)	7,140 (83.2%)
	Mean identity percent	56.6%	74.5%
	Max alignment size	54,158	58,656
	Error-free reads	0	0
NaS reads aligned using BWA mem	# reads	4,717	6,558
	# reads (>10Kb)	167	3,045
	Cumulative size (Mbp)	17.2	65.6
	Average size (bp)	3,639	10,008
	N50 size (bp)	4,273	12,685
	Max size (bp)	31,283	59,863
	Aligned reads	4,717 (100%)	6,558 (100%)
	Mean identity percent	99.9937%	99,9893%
	Max alignment size	31,283	59,863
	Error-free reads	4,620 (97.9%)	6,307 (96.2%)

### NaS overview

**Figure 2:**
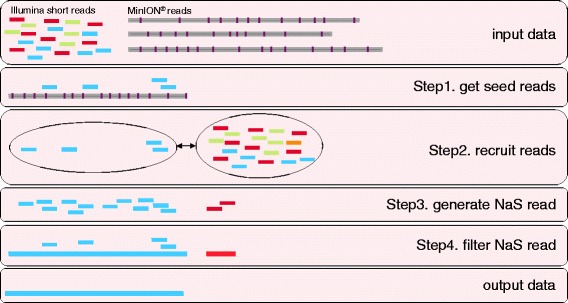
The NaS workflow. Inputs are the Illumina short reads and the MinION® reads (1D and 2D), purple bars on MinION® reads represent sequencing errors. Step1. Illumina reads are aligned on the MinION® templates to select *seed*-*reads* (light blue rectangles). Step2. *Seed*-*reads* are used to recruit similar reads in the initial Illumina read set. Step3. Good recruits (i.e., reads coming from the right genomic region) are light blue rectangles, bad recruits (i.e., reads coming from another similar genomic region) are red rectangles. Step4. Overlap-layout-consensus-based assembly of the *recruited-reads* and the *seed-reads*. Outputted contigs (light blue and red rectangles) are then filtered using *seed-read* alignments. In this example, after filtering step, a single contig representing the final NaS read is produced.

Because the accuracy of MinION® reads is not high (more than 30% of errors), we developed the NaS workflow to overcome the limitation of existing assemblers. The ability to successfully align Illumina reads on MinION® templates is strongly reduced and as a result we observed that existing methods like proovread [13] are not performing well with this new type of data (see Methods). Instead of using Illumina short reads to correct MinION® reads, we propose a method that uses the MinION® read as a template to recruit Illumina reads and, by performing a local assembly, build a high-quality synthetic read (Figure 2). In the first step, a stringent alignment is performed to efficiently retrieve Illumina short reads and their complementary sequences, called *seed*-*reads*. Next, the *seed-read* set is extended by searching for similar reads and their complementary sequences in the initial set (see methods). This second step is crucial to retrieve Illumina reads that correspond to low-quality regions of the template (Additional 1: Figure S1). Finally, a micro-assembly of the reads is performed, using an overlap-layout-consensus strategy (see Methods).

In most cases (99.2% of the 66,492 MinION® reads described below), none or one contig is obtained per MinION® template. However in repeated regions, the micro-assembly leads to a complicated *contig-graph* structure. In fact, in the second step of the process, a small fraction of reads that did not come from the correct genomic regions were recruited. These incorrect reads produce contigs, named *foreign-contigs* that should not be associated with the MinION® template; moreover these contigs generate branch points in the *contig-graph*.

**Figure 3:**
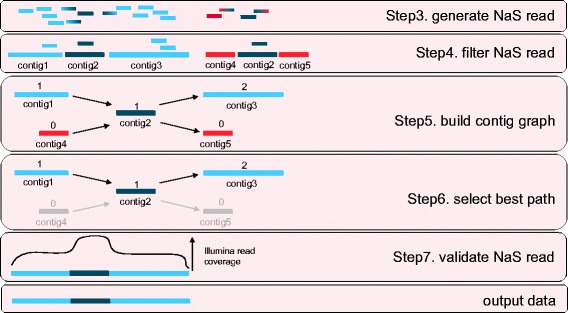
Untangling complex regions. In the case of repetitive regions (represented by dark blue rectangles), the NaS workflow produced several contigs per MinION® template (Step3 and Step4). Indeed, the NaS read is fragmented, due to the indeterminate position of the repetitive region, contig2. Step5. Construction of the contig graph weighted with the *seed-reads* coverage of the given contig. Contig2, which represents the repetitive region, is linked to four different contigs. Step6. The contigs present in the path with the highest weight (contig1 – contig2 – contig3) are selected, using the Floyd-Warshall algorithm, and assembled to generate the final NaS read. Step7. The consistency of the synthetic NaS read is checked by aligning the initial Illumina reads set and detecting gap of coverage.

The basic idea to solve the repeats problem and to remove *foreign-contigs* from the assembly was to select the path that used the contigs with the highest *seed-reads* coverage (Figure 3). Then the consistency of the output synthetic read was checked by aligning the initial Illumina reads set.

We chose a micro-assembly strategy, instead of a classical polishing of the consensus, which would have been more error prone to precisely place Illumina reads on the MinION® template because of the high error rate. One major drawback of the micro-assembly approach is the generation of potential chimeric reads. To overcome this limitation, we developed, as previously described, a specific approach based on a graph traversal, and we added a validation step at the end of the process.

The whole NaS workflow is easy to parallelize, as the processing is the same for each input MinION® template. We used the shell tool, GNU parallel [15], for executing jobs. The elapsed time was between 30 min and 3 h for each dataset. For instance, the NaS reads from the 2D reads of the MinION® run2 were produced on a 16-core computer in 34 min. The average CPU time is less than 1 min per NaS read on a single core computer.

### Impact of Illumina coverage and read length on NaS reads

By sampling randomly the initial Illumina dataset (obtained from a 530- to 630-bp fragment library of the bacterium *Acinetobacter baylyi ADP1* genomic DNA), we generated subsets from 10 × to 150 × genome coverage. Furthermore, we trimmed reads to obtain subsets with the corresponding read length: 100 bp, 150 bp, 200 bp, 250 bp and 300 bp. Interestingly, we found that NaS reads were of high quality, even with a coverage as low as 20 × (Additional 1: Figure S2). Additional coverage may be used to generate longer NaS reads, but the method reached a plateau rapidly. For instance, the average size increase of only 4% between 30 × and 150 × when considering 200 bp Illumina reads. Likewise, the error rate was not sensitive to the coverage, and remained above 99.99%. Strikingly, the optimal read length was 200 bp and not the longer one. It could be a consequence of the initial size of the library (650- to 750-bp) and the lower accuracy of bases located at the end of Illumina reads. Indeed, the 250 bp and 300 bp sequencing generate more overlapping direct and reverse reads. These results demonstrated that our method could be used using Illumina MiSeq reads (2 × 300 bp) as well as Illumina HiSeq 2500 (2 × 250 bp) reads in the case of larger genomes, to drop off the cost.

### *Acinetobacter baylyi ADP1* dataset

**Figure 4:**
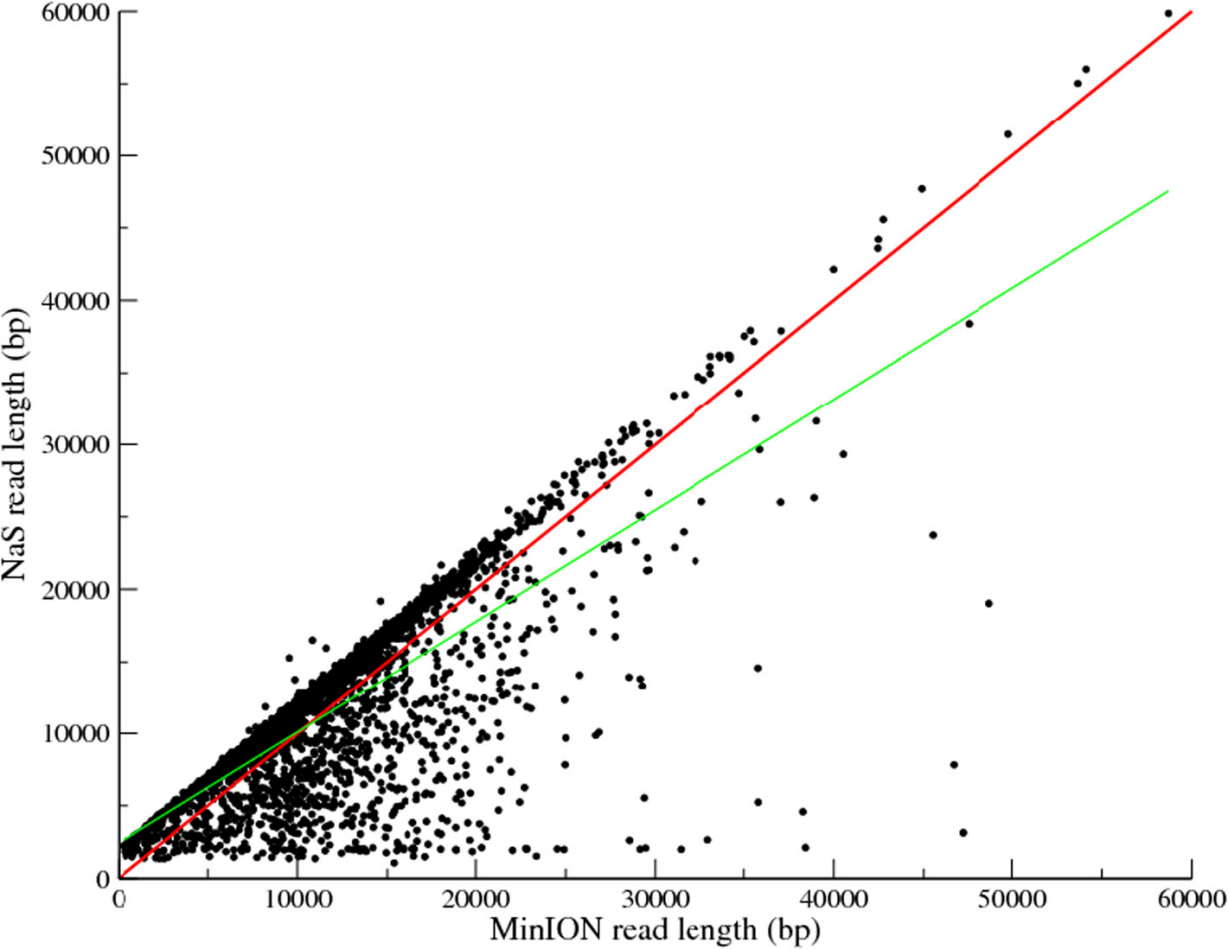
Comparison of MinION® and NaS 2D read length. The x axis represents the 2D MinION® read lengths from run5, and the y axis the length of resulting NaS reads. The red line represents x = y, and the green line shows the linear regression.

To validate our approach, we used our five MinION® runs from the bacterium *Acinetobacter baylyi ADP1.* NaS was launched using several subsets of Illumina reads and those corrected using 50 × of coverage with 250 bp reads were kept (corresponding to the subset which maximizes the coverage of the reference sequence). We applied the NaS approach using the previously described 66,492 MinION® reads and it generated 11,275 NaS reads (with a cumulative size of 82.8 Mb, a N50 of 11,292 bp, and a longest read size of 59,863 bp, Table 2). Only 17% of the initial MinION® templates lead to a NaS reads, this low success rate is directly correlated with the error rate, indeed this is in agreement with the number of reads we were able to map onto the reference genome (25.6%, Table 2). Moreover, we observed a higher success rate with 2D reads (76.4%) compared with 1D reads (8.1%). In contrast with correction-based methods, 62.3% of NaS reads are longer than their corresponding 2D MinION® templates owing to recruitment of reads outside the border of the template. In this case, the 6,558 NaS reads are on average 1,670 bp longer than their corresponding 2D MinION® template (Figure 4). This elongation size relies first on the recruit-step of the NaS workflow, which retrieves similar reads outside the MinION® template, and second on the fragment size of the Illumina library. We observed a higher number of longer NaS reads when generated from the 2D reads compared with when NaS reads were generated from the 1D reads (62.3% for 2D vs 18.7% for 1D, Additional file 1: Figure S3). This can be explained by the lower error rate in the 2D reads (25.5% for 2D vs 43.4% for 1D, Table 2) that makes *seed-read* capturing easier.

To inspect the quality of the NaS reads, they were aligned to the reference genome using BWA mem aligner [16]. The 11,275 NaS reads cover 99.96% of the reference genome and align with an average identity of 99.99%. Ninety-seven percent of the reads align completely with the reference with no error and 99.2% align when allowing one error. Furthermore, the four NaS reads longer than 50 kb aligned perfectly with no error on the reference genome. Two different regions of the reference genome are not covered, implying two gaps of the following size: 1076 bp and 408 bp. We observed that these two genomic regions contain repeated elements. When comparing the coverage distribution of NaS and MinION® reads, we observed that most of the genome is covered accordingly by the two dataset (Additional 1: Figure S4). However, we observed in few cases, a lower coverage in NaS reads. These regions of low coverage mostly contain repeated elements. Generally, if the input MinION® read do not span entirely the repeated element, NaS workflow is not able to generate a read of the same length than the MinION® template. As a consequence, the coverage of large repetitive regions (> 1 Kb) is lower (12.6 in average) than non-repetitive genomic regions (18.4 in average).

The genome of *A. baylyi* harbors seven scattered rDNA clusters, four of which are identical (rDNA clusters 1, 2, 4, and 7). Fourty-three NaS reads spanned completely the seven rDNA clusters and include neighbour sequences that can anchor a read to its true location. Each rDNA cluster is spanned completely by the following number of NaS reads: 9, 10, 2, 4, 7, 4 and 7 respectively. For instance, rDNA cluster 1 is spanned by a NaS read of 19,726 bp (10 kb and 4 kb anchored to the left and right of the cluster, respectively) that aligned entirely and with an identity percent of 99.99%, presenting only two mismatches (Additional 1: Figure S5).

### Genome assembly

**Figure 5:**
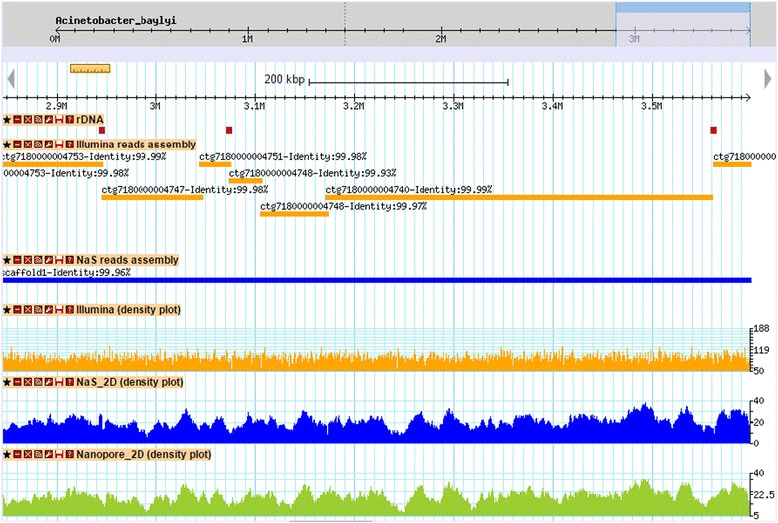
Comparison of Illumina and NaS reads assemblies. The figure shows a capture of a 700 kb genomic region from *Acinetobacter baylyi ADP1*. The first track contains rDNA clusters 5, 6 and 7 (purple rectangles). The orange rectangles represent alignments of contigs from the Illumina-only assembly, whereas blue rectangle represents the alignment of the NaS assembly contig. The three plots represent respectively the coverage of Illumina, Nas 2D and MinION® 2D reads. We observed that breakpoints of the Illumina assembly coincide in part with rDNA clusters, in contrast with the NaS assembly which exhibits a perfect alignment.

To demonstrate the utility of the NaS workflow, we attempted a NaS reads assembly using the Celera assembler [17] and the set of 11,275 NaS reads previously described, representing a 23 × genome coverage. Our assembly was initially composed of 3 contigs, compatible with the two regions devoid of NaS reads. We then used the input MinION® reads with the SSPACE-LongRead [18] scaffolder to produce the final assembly, which is composed of a single scaffold. The 3.6 Mb sequence covered 99.8% of the reference genome with an identity greater than 99.98%. To evaluate the advantage of using the NaS reads for assembly, we performed a control assembly based on the subset of 50 × Illumina 250 bp PE reads using the Celera assembler. Although this assembly harbors a high continuity (20 contigs with a N50 size of 326 kb) and a good completion (99.7% of the reference genome is covered), no contigs were found that spanned a rDNA cluster (Figure 5). The fact that NaS reads are able to go through complex and repetitive regions explains why assemblies based on NaS reads lead to higher quality in terms of solving repeat regions.

We performed two other assemblies by lowering the input coverage, and used respectively 14.4 × and 28.6 × of MinION® reads (respectively 5.3 × and 10.6 × of NaS reads). The final assemblies, respectively composed of 19 and 5 scaffolds, were still less fragmented than the illumina-only assembly (Table 3). This last result showed that even with a low coverage of MinION® reads, the result obtained is still valuable.

**Table 3:** **Comparative statistics of assemblies generated from subsets of NaS or Illumina reads**

	**Assembly 1**	**Assembly 2**	**Assembly 3**	**Illumina_only assembly**
MinION® coverage	14.4x	28.6x	57.0x	NA
Illumina	50x @250 bp	50x @250 bp	50x @250 bp	50x @250 bp
NaS coverage	5.3x	10.6x	23.0x	NA
# of scaffolds	19	5	1	20
Cumulative size (Mbp)	3,431,926	3,599,306	3,600,135	3,592,537
Average size (bp)	180,628	719,861	3,600,135	179,627
N50 size (bp)	242,347	1,815,485	3,600,135	326,117
L50	4	1	1	5
N90 size (bp)	83,428	421,811	3,600,135	140,386
L90	14	3	1	11
Max size (bp)	755,415	1,815,485	3,600,135	520,993
Genome fraction (%)	92.855	99.551	99.880	99.735
# misassemblies	4	2	1	4
# mismatches per 100 kbp	6.46	4.97	4.67	6.49
# indels per 100 kbp	4.64	3.27	3.20	0.33

This sequencing strategy is easy to set up and manage, even in a time-constrained framework. The PE Illumina and MinION® libraries were prepared in 6 and 3 h respectively, the sequencing was spread over 2 days (48 h for both the MiSeq 2*300 PE run and for one MinION® run) and the computational step (NaS workflow and genome assembly) is no longer than 24 h on a standard 16-cores computer (15 h for NaS workflow and 5 h for the genome assembly step).

## Conclusion

The approach we present here is an efficient method to sequence genome by combining advantage of Illumina and the new Oxford Nanopore technologies. These sequencing technologies are commercialized through two desktop instruments, the MinION® device and the MiSeq sequencer respectively, that have the advantage to be small and relatively low cost. Our method, based ideally on at least 20 × and 50 × of NaS and Illumina reads respectively, offers the opportunity to sequence microbial or small eukaryotic genomes in a very short time, even in small facilities, to high accuracy with informatics finishing steps. This hybrid approach presents an interesting alternative compared with standard strategies, such as SMRT of Pacific BioSciences and Illumina TruSeq Synthetic long reads. For example, our approach is straightforward in terms of library preparation, as well as laboratory and information technology infrastructure requirements. The real novelty is to give access to these accurate genome assemblies through desktop and portable sequencers. The limitation of our method is currently the throughput of the MinION® device, however if a higher throughput of the Oxford Nanopore technology becomes available, it may be speculated that NaS would provide an efficient method for sequencing organisms with large and repetitive genomes. Finally, this study shows that although the Oxford Nanopore technology is a relatively new sequencing technology, currently with a high error rate, it is already useful in the generation of high-quality genome assemblies with an adapted strategy.

## Methods

### DNA extractions

*Acinetobacter baylyi ADP1* genomic DNA was prepared from overnight liquid cultures grown in MAS (Medium for *Acinetobacter* Supplemented) broth at 30°C with shaking to an O.D._600_ of approximately 1.5. Cells were pelleted and lysed in the presence of Lysozyme from chicken egg white (Sigma, St. Louis, MO, USA). Genomic DNA was purified by phenol-chloroform (Phenol-chloroform-isoamyl alcohol mixture, Sigma) phase extraction. Extracted DNA was resolved in 100 μL TE buffer (10 mM Tris, 1 mM EDTA [pH 8.0]) supplemented with 10 μg/mL RNase (Sigma).

### Illumina library preparation and sequencing

DNA (30–100 ng) was sonicated to a 100- to 800-bp size range using a Covaris E210 sonicator (Covaris, Woburn, MA, USA). Fragments were end-repaired, 3′-adenylated and Illumina adapters were then added using the NEBNext Sample Reagent Set (New England Biolabs, Ipswich, MA, USA). Ligation products were purified using Ampure XP (Beckmann Coulter Genomics, Danvers, MA, USA) and DNA fragments (>200 bp) were PCR amplified using Illumina adapter-specific primers and Platinum Pfx DNA polymerase (Invitrogen, Carlsbad, CA, USA). Amplified library fragments of 650–750 bp were size selected on a 3% agarose gel. Libraries were quantified by qPCR using the KAPA Library Quantification Kit for Illumina Libraries (KapaBiosystems, Wilmington, MA, USA) and library profiles were assessed using a DNA High Sensitivity LabChip kit on an Agilent Bioanalyzer (Agilent Technologies, Santa Clara, CA, USA). Libraries were sequenced on an Illumina MiSeq instrument (San Diego, CA, USA) using 300 base-length read chemistry in a paired-end mode.

### Nanopore 8 kb and 20 kb libraries preparation

*Acinetobacter baylyi* genomic DNA was sheared using G_Tubes (Covaris) according to the following conditions: i) for 8 kb library: 5 μg of genomic DNA in 150 μl Elution Buffer (EB, Tris HCl 10 mM; Qiagen, Hilden, Germany) was centrifuged in a G-Tube at 3.3 × g for 1 min before inverting the tube and centrifuging again for 1 min; ii) for 20 kb library: 10 μg of genomic DNA in Elution Buffer was loaded in six G-Tubes (100 μl aliquots each) and centrifuged at 1.1 × g for 1 min before inverting the tubes and centrifuging again for 1 min. Eight kb fragmentations were evaluated using a DNA12000 LabChip kit on an Agilent Bioanalyzer and quantified using a Qubit Fluorometer (Life Technologies, Carlsbad, CA, USA). Then, one microgram of 8 kb sheared DNA was end repaired in 100-μL reactions using the NEBNext End-Repair module (New England Biolabs) according to the manufacturer’s instructions. For 20 kb libraries, two pools of fragmented DNA were created, each containing DNA from three fragmentations. The two preparations were cleaned-up using 0.4 × AMPure XP beads, eluted in 80 μL EB and quantified using a Qubit Fluorometer. Two end repair reactions, containing around 2 μg fragmented DNA, were performed using the NEBNext End-Repair module (New England Biolabs).

All end repair reactions were cleaned-up using 1 × AMPure XP bead purification according to the manufacturer’s instructions (Beckmann Coulter Genomics) and eluted in 28 μL EB. For 8 kb and 20 kb libraries respectively, one and two A-tailing reactions were performed on 25 μL of the DNA using the NEBNext dA-tailing module (New England Biolabs) in a total volume of 30 μL according to the manufacturer’s instructions.

The Genomic DNA Sequencing Kit, SQK-MAP-002 (Oxford Nanopore Technologies Ltd, Oxford, UK), was used to generate MinION® sequencing libraries. Fifty microliters of Blunt/TA ligase master mix (New England Biolabs), 10 μL adapter mix, and 10 μL HP adaptor were added to each dA-tailed DNA and incubated at 20°C for 10 min*.* For the 20 Kb library, the ligation reactions were pooled, and the 8 kb and 20 kb libraries were cleaned up using 0.4 × volumes of AMPure XP beads according to the manufacturer’s instructions, with the exception that only a single wash was carried out using the wash buffer supplied with the kit. The samples were then eluted in 25-μL elution buffer supplied with the Genomic DNA Sequencing Kit. Ten microliters of *tether* (Genomic DNA Sequencing Kit) was added and incubated for 10 min at 20°C. Last, 15 μL of *HP motor* (Genomic DNA Sequencing Kit) was added and incubated overnight at 20°C, giving a total library volume of 50 μL.

### MinION™ Flow Cell preparation and sample loading

For each run, a new MinION™ Flow Cell was removed from storage at 4°C, fitted to the MinION® device and held in place with the supplied plastic screws to ensure a good thermal contact. One hundred and fifty microliters of EP Buffer (Genomic DNA Sequencing Kit) was loaded into the sample loading port and left for 10 min to prime the flowcell. The priming process was repeated a second time. Then, for every prepared library (single tube for the 8 kb library or pool of the two tubes for the 20 kb library), 12 μL of library and 4 μL of Fuel Mix (Genomic DNA Sequencing Kit) were added to 136 L of EP Buffer (Genomic DNA Sequencing Kit) and loaded into the sample loading port of the MinION® Flow Cell. The loading was repeated three times: 6, 24, and 30 h after the beginning of the run.

### MinION® sequencing and reads filtering

Read event data generated by MinKNOW™ control software (version 0.45.3.9) were base-called using the software Metrichor™ (version 0.17). The data generated (pores metrics, sequencing, and base-calling data) by MinION® software are stored and organized using a Hierarchical Data Format (HDF5). Three types of reads were obtained: template, complement, and two-directions (2D). Template and complement reads correspond to sequencing of the two DNA strands. Metrichor™ combines template and complement reads to produce a consensus (2D) [9]. FASTA reads were extracted from MinION® HDF5 files using poretools [19]. To assess the quality of the MinION® reads, we aligned reads against the *A. baylyi ADP1* reference genome using the LAST aligner (version 460) [20]. As the MinION® reads are long and have a high error rate we used a gap open penalty of 1 and a gap extension of 1.

### Illumina reads processing and quality filtering

After Illumina sequencing, an in-house quality control process was applied to reads that passed the Illumina quality filters. The first step discards low-quality nucleotides (Q < 20) from both ends of the reads. Next, Illumina sequencing adapters and primers sequences were removed from the reads. Then, reads shorter than 30 nucleotides after trimming were discarded. These trimming and removal steps were achieved using in-house-designed software based on the FastX package [21]. The last step identifies and discards read pairs corresponding to the PhiX genome, using SOAP [22] and the PhiX reference sequence (NC_001422.1). This processing results in high-quality data and improvement of subsequent analyses.

### Test of correction-based approach

We applied proovread [13], a recently available correction tool, to our MinION® reads. We limited our benchmark to the 2D reads of run2, with 100 × of Illumina PE reads. The results showed that correction-based methods do not function satisfactorily with this new type of data. Indeed, proovread produced a corrected version for 344 of the 543 MinION® input reads. However, when mapping these corrected reads to the reference genome using BWA mem [16] with the “-x pacbio” parameter, we were able to aligned only 63.35% of the 344 corrected reads, and we computed an average identity percent of 71.6%, which is not different from that obtained when aligning non-corrected reads (67.2%) using the same software and parameters (Additional file 1: Table S2).

### The NaS pipeline

In a first step, the Illumina reads were aligned on the MinION® templates using BLAT [23] with the following parameters: tileSize = 10 and stepSize = 5. We retrieved missing reads with Commet [24] using the *seed-reads* previously obtained as probe and the initial Illumina reads set as target. A given read is considered similar to a *seed-read* and then retrieved, if they share several common k-mers. For stringent recruiting we used the following parameters: three non-overlapping 32-mers (−t 3 and –k 32). The *seed-reads* and recruited reads were assembled using an OLC approach through Newbler v2.9 with the following parameters: −urt (to avoid contig breaks in low-covered regions) and -mi 98 (for stringency and to take advantage of the Illumina read quality). From the whole set of contigs produced by one local assembly, we kept the longest one and those that had a coverage of *seed-reads* greater than MIN_COV1 parameter, we used 10 ×. To compute the contig coverage in *seed-reads*, we filtered out, from the initial BLAT alignment, Illumina reads that aligned on several contigs and/or with low quality alignment (less than 50% of read length or less than 90% of correctly aligned bases).

The Newbler algorithm was unable to solve repeats and broke the contigs around those repetitive regions (Additional 1: Figure S6), as in the Illumina-only assembly. For instance, we observed broken local assemblies in the region of seven rDNA clusters (Additional 1: Figure S7). To solve the repeats problem and to remove *foreign-contigs* from the assembly, we built an undirected *contig-graph* based on the Newbler output file “454ContigGraph.txt”. Vertices represent Newbler contigs and edges link between two contigs. Edges are weighted using contig coverage (not coverage of reads used for the assembly, but coverage of *seed-reads*, which represent more reliable reads). The basic idea was to select the path that used the contigs with the highest *seed-reads* coverage. For that purpose, we used the Floyd-Warshall algorithm by negatively scoring edges of the graph. In the case of the seven rDNA clusters of the *A. baylyi ADP1* genome, the algorithm implemented in NaS selected the contig that was built with the highest *seed-reads* coverage from the seven possible source contigs. Repeated contigs and the sink contig were then selected in the same way. Next, we checked the consistency of the output synthetic read by aligning the initial Illumina reads set, using BLAT with the following parameters : tileSize = 12. We invalidated the synthetic read, if we observed a gap in coverage (coverage less than the MIN_COV2 parameter, 10 × was used). If a synthetic read was invalidated, we kept the longest region without drop of coverage (below the given threshold) from this invalid read.

### Alignment programs comparison

The *seed-reads* capturing represents a critical step, indeed for a given MinION® template we need at least one *seed-read* to initiate the NaS workflow. A high sensitivity is needed however the specificity is quite important too. Indeed if too many reads are recruited, the micro-assembly step becomes a whole genome assembly. We compared BWA [16], BWA mem [25], Bowtie2 [26] and BLAT [23] alignment programs performance using 1D and 2D MinION® reads of run2 (Table 1). To overcome the high error rate of MinION® reads, we parametrized each program with a lower seed than the one used by default (Additional file 1: Table S3). In these conditions, we found that BLAT was more sensitive and more computationally efficient than others aligners (Additional file 1: Table S3).

Given their high accuracy, NaS reads were aligned to the reference genome using BWA mem, while MinION® reads were aligned using LAST aligner (Table 2).

### Comparison of Newbler, MIRA and Celera assembler to generate NaS reads

We benched Newbler, MIRA [27] and Celera assembler [17] (CA) on their performance to deal with micro-assembly of synthetic reads. For that purpose, the 543 2D reads of run2 were used (Table 1). The reads recruiting was performed using BLAT [23] and Commet [24] as previously described, and we were able to retrieve Illumina reads for 353 reads of the 543 initial MinION® reads. The three assembly programs (Newbler, MIRA and CA) were launched 353 times, and for each individual assembly the largest contig was kept as the final synthetic read. We observed, in these specific conditions, that Newbler outperformed the two other assemblers (Additional file 1: Table S4). Newbler and CA produced similar results, in terms of number of synthetic reads obtained (352 for CA and 353 for Newbler); in contrast MIRA produced only 242 synthetic reads. The N50 and maximal length of the synthetic reads produced by each program were highly similar, but the quality of synthetic reads is higher with Newbler (Additional file 1: Table S4). Moreover, we showed that Newbler contigs are slightly longer than their CA counterpart (Additional 1: Figure S8). This short elongation is certainly due to the –urt option of Newbler (that avoid contig breaks in low-covered regions as, in this special case, both ends of the synthetic read). One other important aspect is the computational time, as we need to perform numerous micro-assemblies. We observed on our dataset that Newbler is in average 4–5 times faster than MIRA and 18–19 times faster than CA (Additional file 1: Table S4). Finally, Newbler offers a simplify access to the contig graph, through the 454ContigGraph output file which is well-documented. In light of these results, we decided to use the Newbler program to generate NaS reads, although it is not open-source. However, Newbler is freely available at the following URL: http://www.454.com/products/analysis-software/.

### Assemblies and quality assessment

The synthetic NaS reads were assembled separately using the Celera Assembler [17] (CA) with parameters as detailed in Additional file 1: Table S5. Peculiarly, CA produced contigs with ends having a hundred bases of perfect identity. We used minimus2 (with following parameters REFCOUNT = 0; MINID = 99.9; OVERLAP = 500; MAXTRIM = 1000; CONSERR = 0.01) to merge contigs produced by CA. Moreover, one overlap was merged manually. Indeed, minimus2 was not able to fuse two contigs ends which show a near perfect (only one mismatch) 600 bp-overlap. Finally, we used SSPACE-LongRead using the default parameters and the 66,492 MinION® reads, to organize the resulting contigs. Illumina paired-end reads were assembled using the CA with parameters as described in Additional file 1: Table S6. Resulting contigs were aligned to the reference genome using nucmer [28] and quality metrics (genome fraction, misassemblies, mismatches and indels rates) were computed using Quast [29].

### Data accessibility

NaS is freely accessible at http://www.genoscope.cns.fr/nas. The Illumina MiSeq and MinION® data are available in the European Nucleotide Archive under accession number ERP009748. The reference genome of *Acinetobacter baylyi ADP1* is available under the following accession number CR543861.

## Additional file

Additional file 1: All the supporting data are included as a single additional file which contains **Figures S1-S8.** and **Tables S1-S6**.

## Abbreviations

NaS: Nanopore Synthetic-long reads
SMRT: Single-molecule real-time sequencing
MAP: MinION® Access Programme
USB: Universal Serial Bus
