# Supplementary material for: Genome assembly using Nanopore-guided long and error-free DNA reads
Source: BMC Genomics. 2015 Apr 20;16(1):327. doi: 10.1186/s12864-015-1519-z (PMC4460631; doi:10.1186/s12864-015-1519-z)
Supplement: Additional file 1: — All the supporting data are included as a single additional file which contains Figures S1-S8. and Tables S1-S6. [file 12864_2015_1519_MOESM1_ESM.pdf]

Genome assembly using Nanopore-guided Long and Error-free DNA Reads

Mohammed-Amin Madoui[1]*, Stefan Engelen[1]*, Corinne Cruaud[1], Caroline Belser[1], Laurie Bertrand[1], Adriana Alberti[1], Arnaud Lemainque[1], Patrick Wincker[1,2,3], Jean-Marc Aury[1§]

[1]Commissariat à l'Energie Atomique (CEA), Institut de Génomique (IG), Genoscope, BP5706, 91057 Evry, France

[2]Université d'Evry Val d'Essonne, UMR 8030, CP5706, 91057 Evry, France.

[3]Centre National de Recherche Scientifique (CNRS), UMR 8030, CP5706, 91057 Evry, France

*These authors contributed equally to this work

[§]Corresponding author

Supplementary Figures and Tables

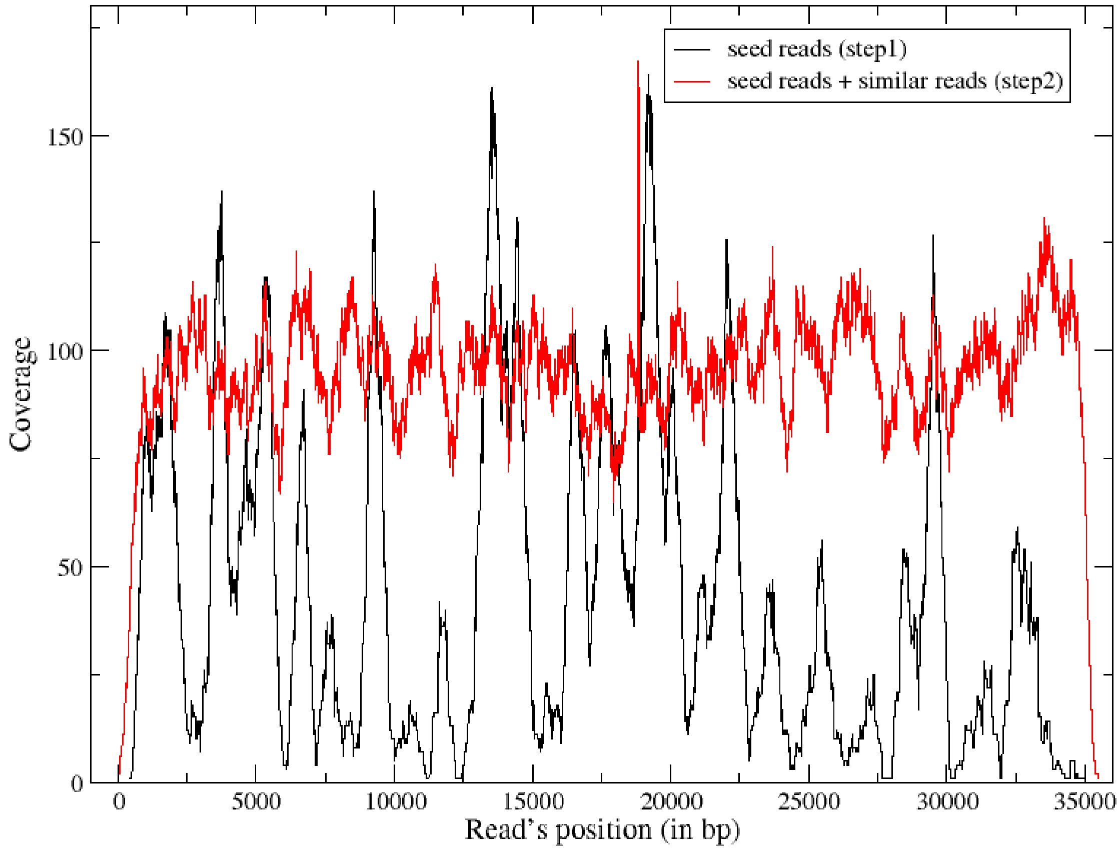

Figure S1. Distribution of Illumina read coverage along a 35-kb MinION® read. The black line represents coverage after the first step (BLAT alignment to find *seed-reads*) and the red line represents coverage after the recruitment step (comparison of *seed-reads* against the whole set of Illumina reads).

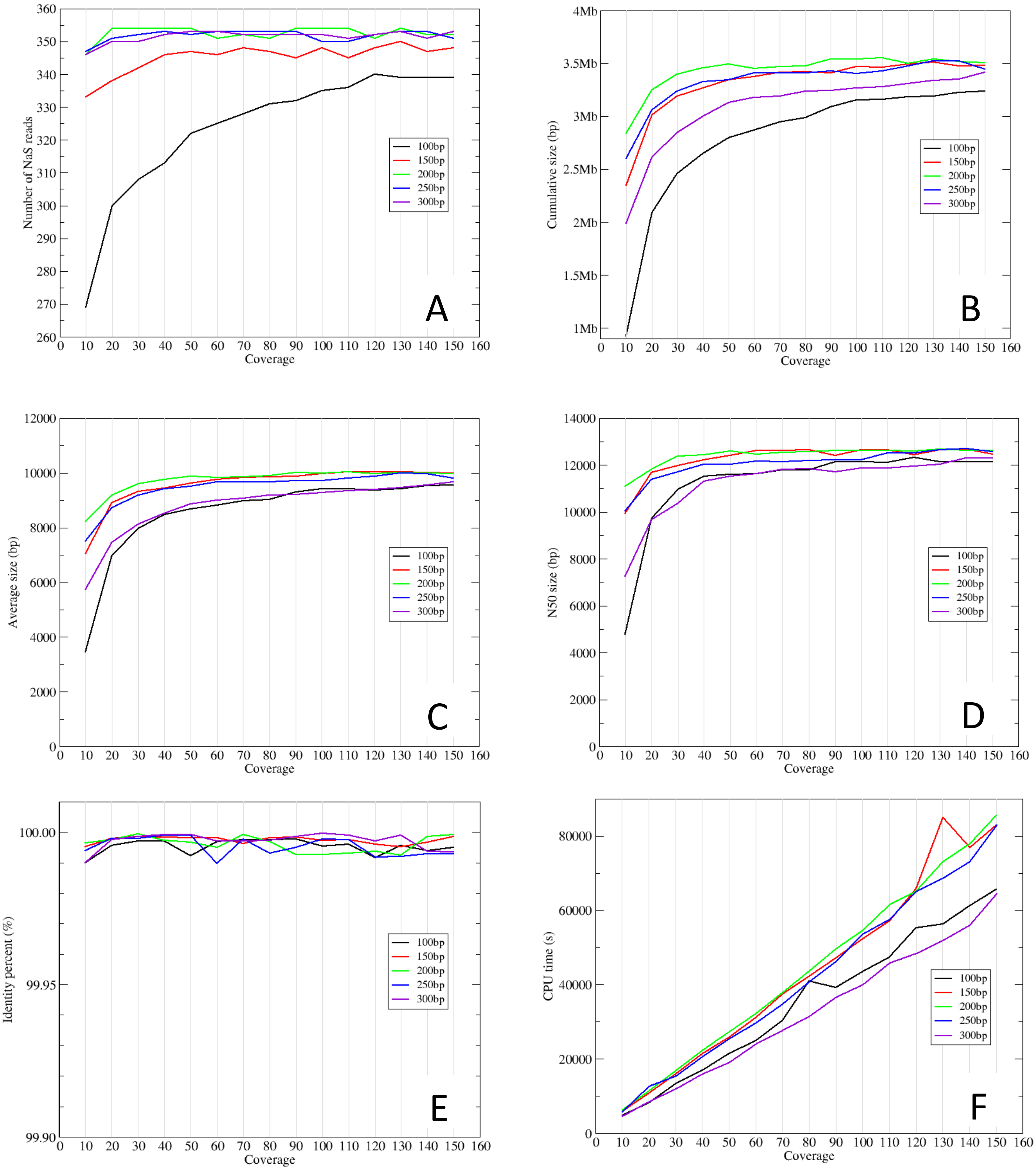

Figure S2. Impact of Illumina coverage and read length on NaS reads. The x-axis represents genome coverage from 10x to 150x. Each curve represents a given read length: 100bp (black), 150bp (red), 200bp (green), 250bp (blue) and 300bp (purple). This benchmark was achieved on 2D reads from run2 (library 20Kb and R7 chemistry). **A.** Number of produced NaS reads. **B.** Cumulative size (in bp) of NaS reads. **C.** Average size (in bp) of NaS reads. **D.** N50 size (in bp) of NaS reads. **E.** Identity of NaS reads aligned to the reference genome. **F.** CPU time (in seconds).

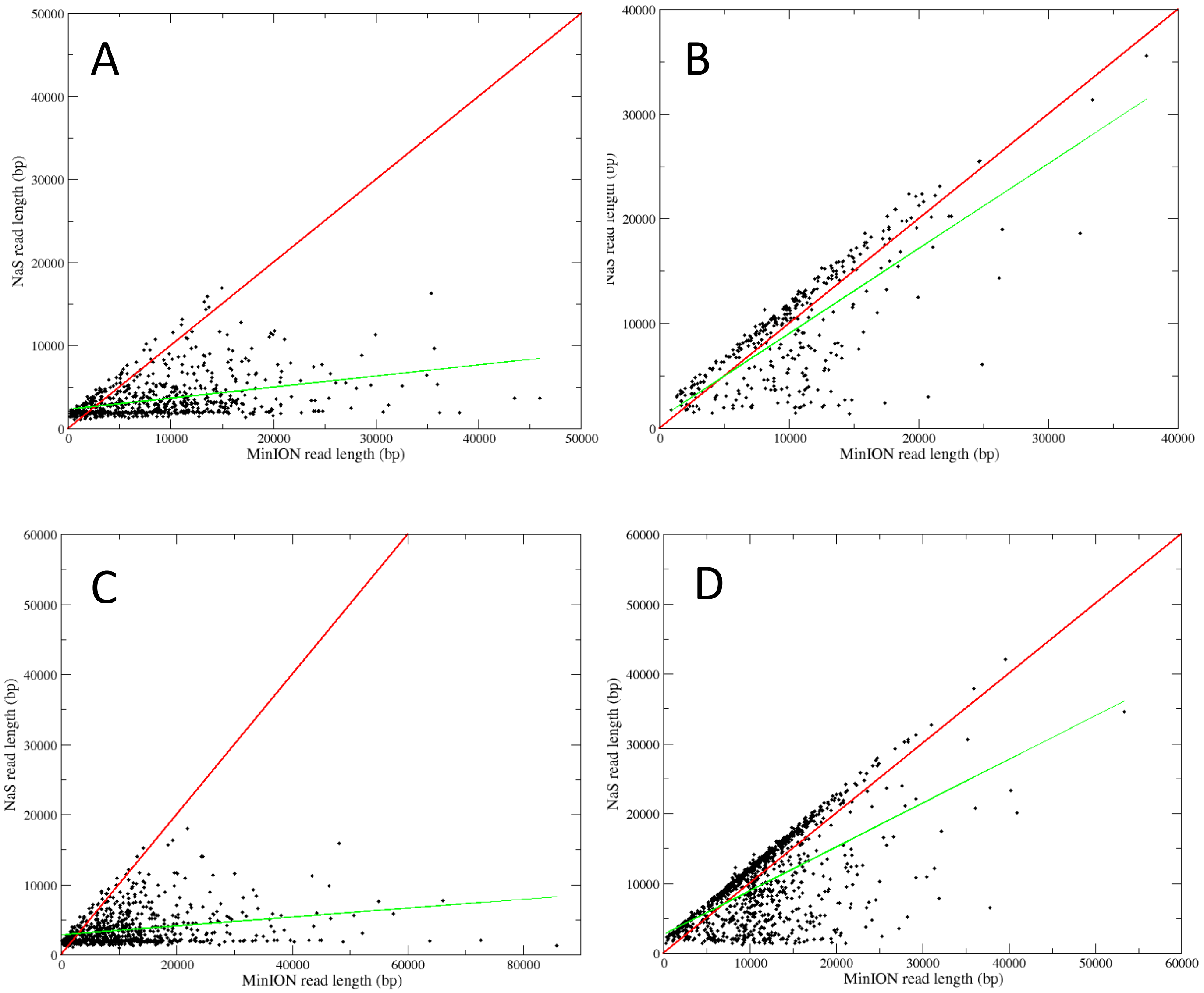

Figure S3. Comparison of MinION® (x axis) and NaS (y axis) read length. Red lines represent x=y, and green lines are linear regressions. **A.** 1D reads from run2 (20-kb library and R7 chemistry). **B.** 2D reads from run2 (20-kb library and R7 chemistry). **C.** 1D reads from run4 (20-kb library and R7.3 chemistry). **D.** 2D reads from run4 (20-kb library and R7.3 chemistry).

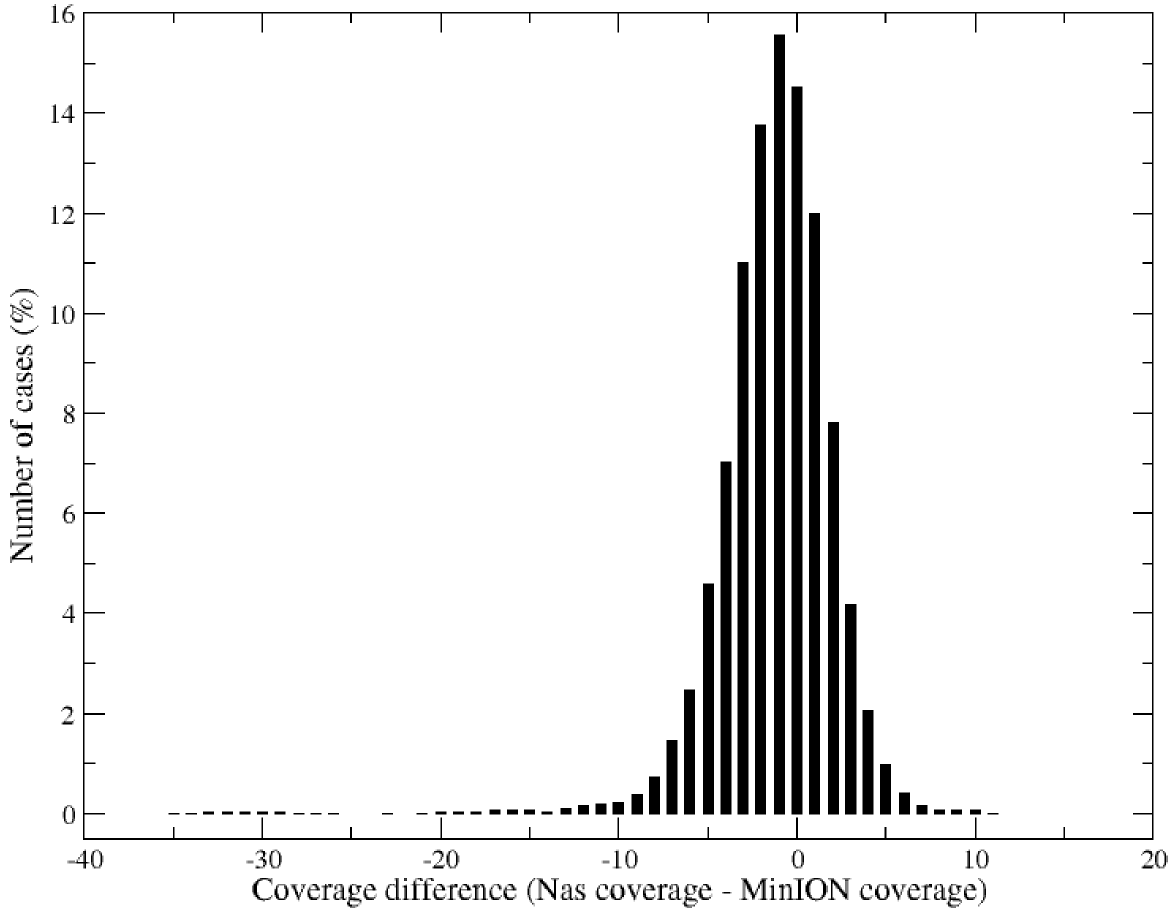

Figure S4. Comparison of MinION® and NaS read coverage. For each base of the reference genome, we computed the difference between coverage in NaS and MinION® reads. A negative value indicates a lower coverage in NaS read, whereas a positive value indicates a higher coverage in NaS read.

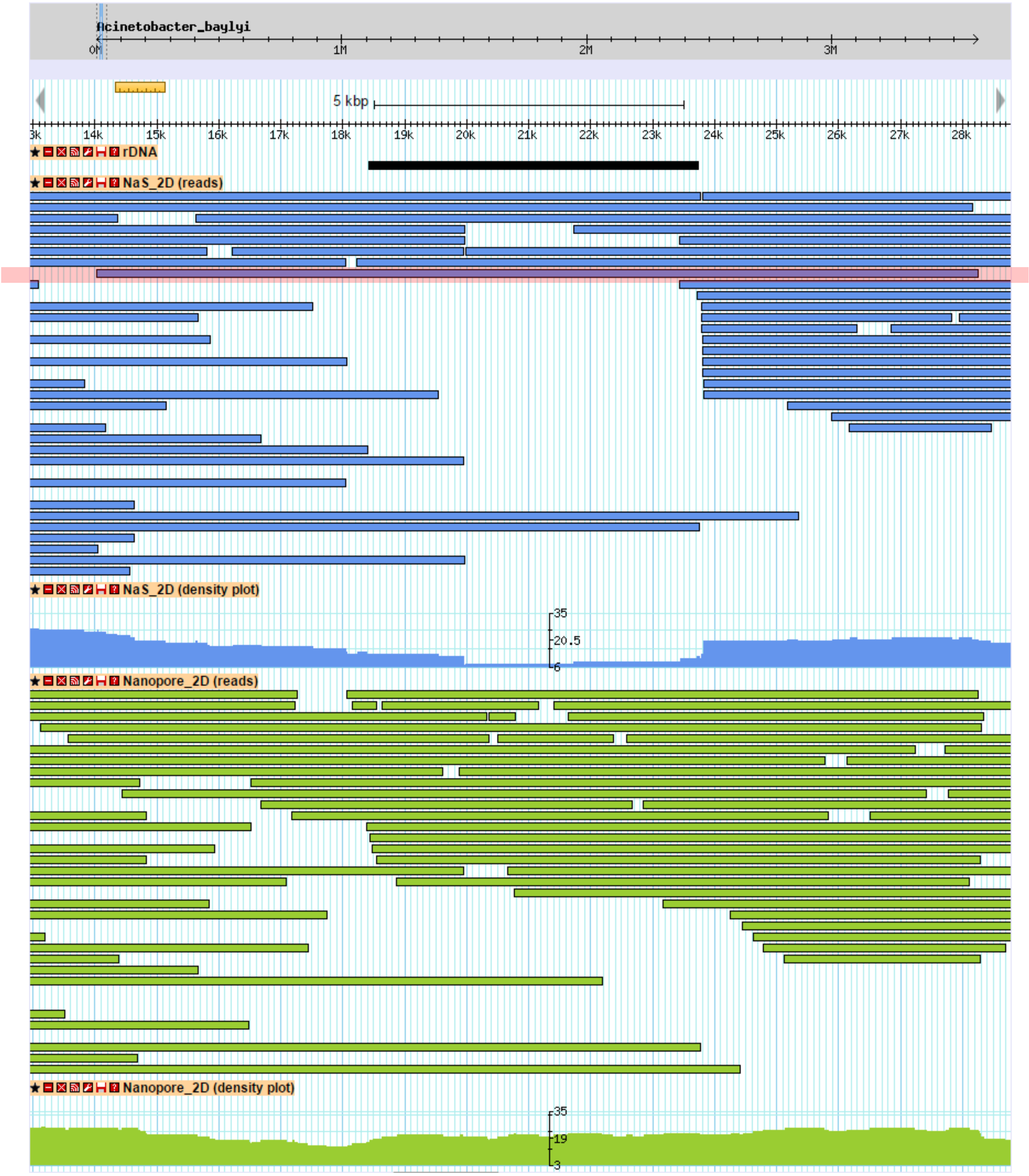

Figure S5. Overview of rDNA cluster 1 of *Acinetobacter baylyi ADP1*. The figure shows a capture of the rDNA1 genomic region from *Acinetobacter baylyi ADP1* (from 18 kb to 24kb). The first track contains rDNA cluster 1 (black rectangle). The blue rectangles represent alignments of 2D NaS reads, whereas green rectangles represent alignments of 2D MinION® reads. The two plots represent respectively the coverage of Nas 2D (blue) and MinION® 2D (green) reads. The 19,726 bp NaS read cited in the main text is surrounded with a red rectangle. As discussed in the main text, we observed a lower NaS read coverage around repetitive region in contrast with the MinION® read coverage.

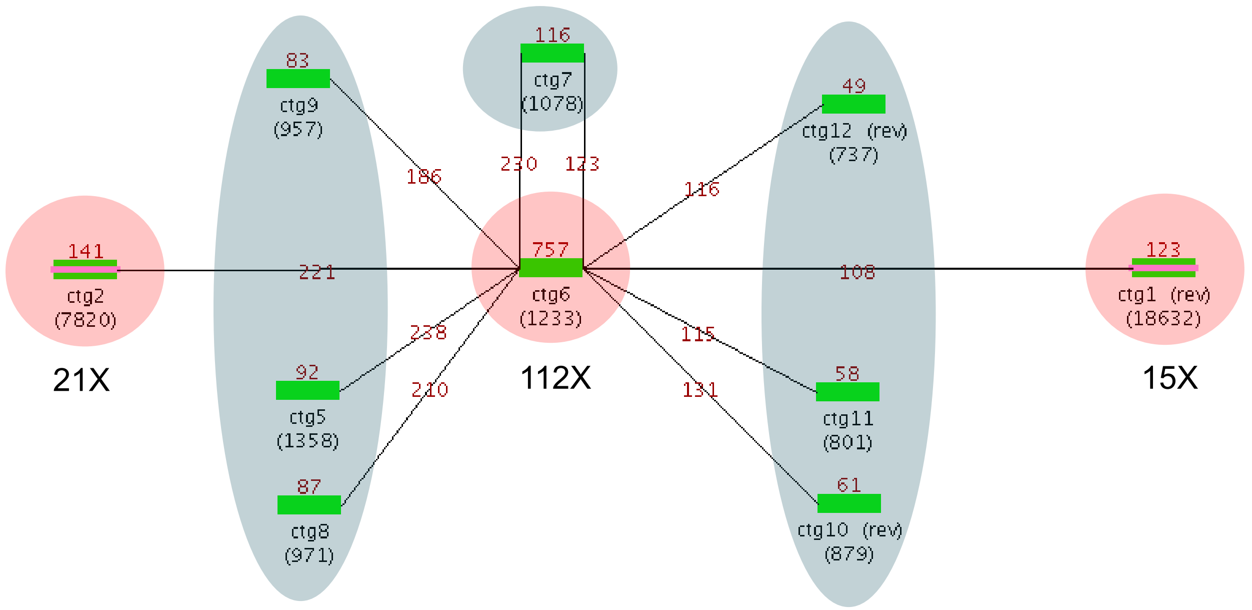

Figure S6. Example of a *contig-graph* generated from a read spanning a repeated region. Contigs encircled in red shading (ctg2, ctg6 and ctg1) are contigs covered by *seed-reads* (coverage is stated in black). Contigs surrounded by blue shading are *foreign-contigs*. Using the Floyd-Warshall algorithm, the following path has been extracted from the graph: ctg2 – ctg6 – ctg1, leading to a 27,685 bp NaS read. Red and black numbers represent respectively the coverage (*seed-reads* and *recruited-reads*) and the length of the corresponding contig.

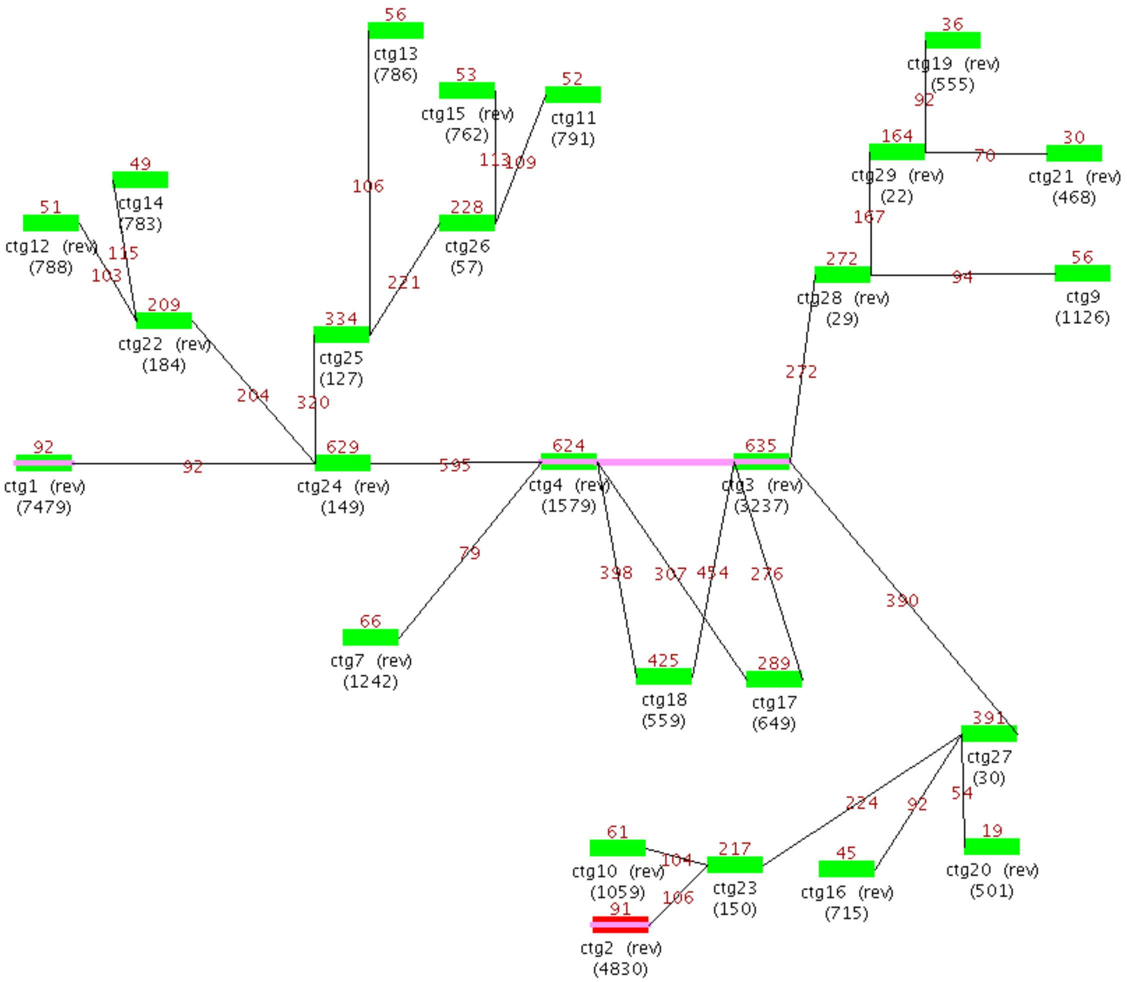

Figure S7. Example of a c*ontig-graph* generated from a read spanning an rDNA locus. Contigs are green boxes; length and coverage of a given contig are respectively the black number in bracket under contig name and the red number over contig name. This graph structure shows 7 sources (ctg1, ctg12, ctg14, ctg13, ctg15, ctg11 and ctg7) and 7 sinks (ctg19, ctg21, ctg9, ctg20, ctg2, ctg16 and ctg10) representing the seven rDNA clusters of *Acinetobacter baylyi ADP1*. Using the Floyd-Warshall algorithm, the following path has been extracted from the graph: ctg1 – ctg24 – ctg4 – ctg17 – ctg3 – ctg27 – ctg23 – ctg2.

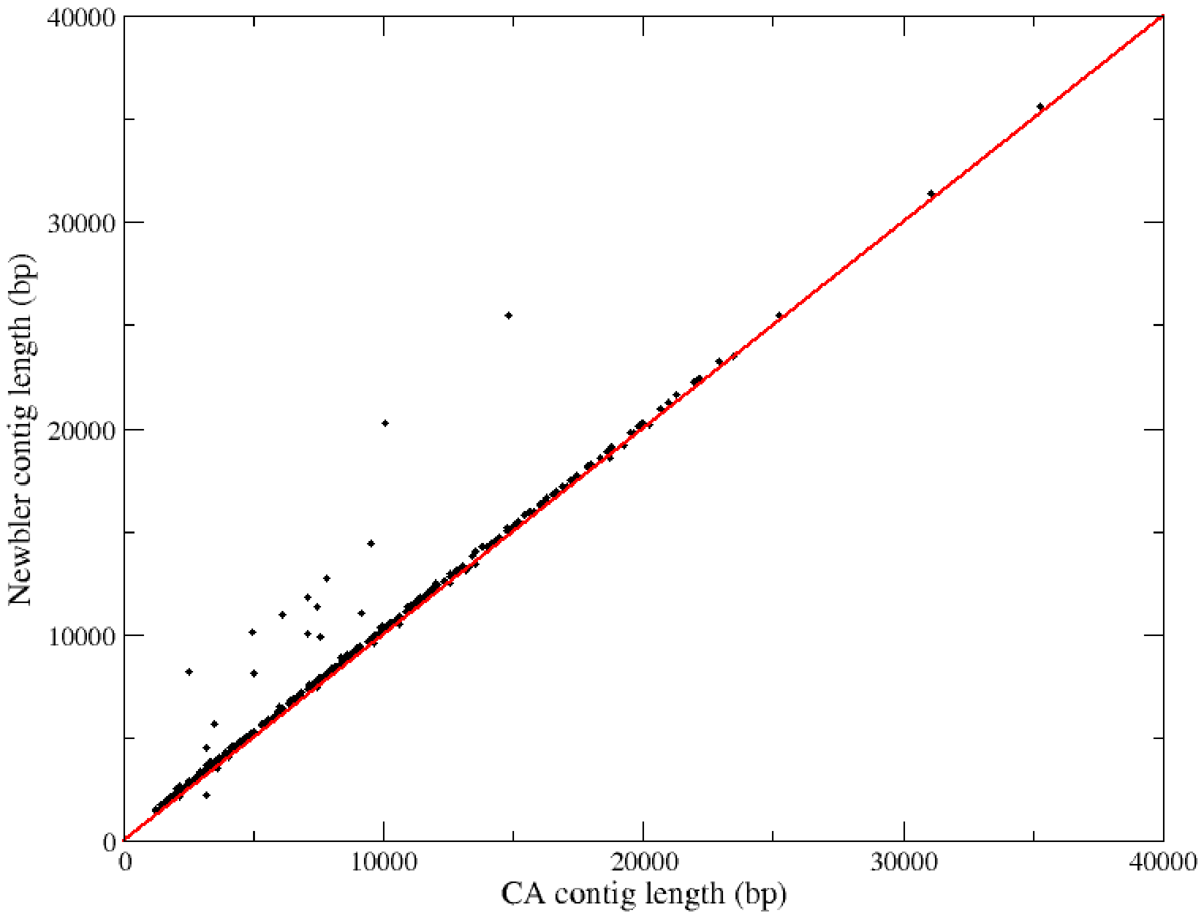

Figure S8.Comparison of Celera assembler and Newbler contig length. The figure shows a comparison of synthetic read size produced using CA (x axis) and Newbler (y axis).

Table S1. Summary statistics of MinION® sequencing (R7 and R7.3 chemistries) and NaS reads

	Read set	R 7		R 7.3	
		1D	2D	1D	2D
MinION® reads aligned using LAST	# reads	12,086	1,145	45,825	7,436
	# reads (>10Kb)	638	275	2,971	3,591
	Cumulative size (Mbp)	32.3	8.3	86.6	77.7
	Average size (bp)	2,672	7,292	1,888	10,455
	N50 size (bp)	6,988	9,641	12,878	12,432
	Max size (bp)	121,776	37,578	123,135	58,704
	Aligned reads	3,451 (28.5%)	870 (76.0%)	6,172 (13.5%)	6,270 (84.3%)
	Mean identity percent	57.30%	68.67%	56.45%	75.05%
	Max alignment size	35,340	36,595	54,158	58,656
	Error-free reads	0	0	0	0
NaS reads aligned using bwa mem	# reads	736	797	3,981	5,761
	# reads (>10Kb)	23	197	144	2,848
	Cumulative size (Mbp)	2.6	5.9	14.6	59.7
	Average size (bp)	3,507	7,396	3,663	10,370
	N50 size (bp)	4,051	9,546	4,306	13,050
	Max size (bp)	16,962	35,576	31,283	59,863
	Aligned reads	736 (100%)	797 (100%)	3,981 (100%)	5,761 (100%)
	Mean identity percent	99.9982%	99.9935%	99.9929%	99.9888%
	Max alignment size	16,962	35,576	31,283	59,863
	Error-free reads	97.1%	97.7%	98.1%	96.0%

Table S2. Comparative summary of MinION® and proovread reads.

	MinION® reads	proovread
# reads	543	543
# reads (>10Kb)	234	234
Cumulative size (Mbp)	5.2	5.2
Average size (bp)	9,615	9,621
N50 size (bp)	11,226	11,273
Max size (bp)	37,578	36,598
Aligned reads	344 (63.35%)	344 (63.35%)
Mean identity percent	61.611%	71.5677%
Max alignment size	36,623	35,643
Error-free reads	0	0

TableS3. Performance comparison of BLAT, BWA, BWA mem and Bowtie2 programs.

	Assembly programs	BLAT	BWA	BWA mem	Bowtie 2
	Version	36	0.7.10	0.7.10	2.2.4
	Parameters	tileSize=10 stepSize = 5	-l 10 –k 0	-k 10	-N 0 –L 10
3,477 1D reads	CPU time (seconds)	654	39	3,986	1,155
	Number of MinION reads with at least one hit	745	0	336	1
543 2D reads	CPU time (seconds)	578	26	2,525	1,060
	Number of MinION reads with at least one hit	353	4	325	50

Table S4. Performance comparison of Newbler, MIRA and Celera assembler (CA). Final contigs were aligned using BWA mem software.

Assembly programs	Newbler	MIRA	CA
Parameters	-urt –mi 98	job = genome,denovo,accurate technology = solexa	ovlHashBits = 25 ; cnsMinFrags = 1 ; ovlErrorRate = 0.1 ; cnsErrorRate = 0.1 ; utgErrorRate = 0.1 ; utgGraphErrorRate = 0.1
# reads	**353**	242	352
# reads (>10Kb)	**148**	117	135
Cumulative size (Mbp)	**3.30**	2.7	3.14
Average size (bp)	9,368	**11,175**	8,912
N50 size (bp)	11,790	**11,947**	11,473
Max size (bp)	**35,585**	34,954	35,288
Aligned reads	**353 (100%)**	**242 (100%)**	**352 (100%)**
Mean identity percent	**99.9998%**	99.9891%	99.9982%
Error-free reads	**349 (98.86%)**	93 (38.42%)	325 (92.32%)
Coverage of the reference	**59.2%**	52.0%	57.2%
Average CPU time (seconds)	**5.6**	26	105
Cumulative CPU time (seconds)	**566**	2,603	10,947

Table S5. Celera assembler parameters used to assemble NaS reads.

merSize	17
merThreshold	0
merDistinct	0.9995
merTotal	0.995
doOBT	1
unitigger	bogart
ovlErrorRate	0.1
utgGraphErrorRate	0.1
utgMergeErrorRate	0.1
cnsErrorRate	0.1
cgwErrorRate	0.1
ovlConcurrency	24
cnsConcurrency	24
ovlThreads	1
ovlHashBits	22
ovlHashBlockLength	10000000
ovlRefBlockSize	10000
cnsReuseUnitigs	1
cnsMinFrags	100
cnsPartitions	64

Table S6. Celera assembler parameters used to assemble Illumina reads.

ovlConcurrency	4
ovlThreads	8
ovlCorrConcurrency	20
ovlCorrBatchSize	1000
ovlHashBits	25
ovlRefBlockSize	4000000
ovlHashBlockLength	100000000
cnsConcurrency	48
cnsMinFrags	100
frgCorrConcurrency	20
frgCorrBatchSize	1000
frgCorrThreads	4
frgCorrOnGrid	1
merylThreads	20
merylMemory	100000
mbtConcurrency	20
mbtThreads	4
ovlErrorRate	0.02
utgGraphErrorRate	0.01
merOverlapperExtendConcurrency	12
merOverlapperSeedConcurrency	12
merOverlapperThreads	4
utgGenomeSize	3600000
unitigger	bogart
